# Diagnostic difficulties of primary angiosarcoma of the breast: a case report

**DOI:** 10.1186/s13256-018-1772-2

**Published:** 2018-08-22

**Authors:** Youssef Mahdi, Lamiaa Rouas, Abderrahmane Malihy, Najat Lamalmi, Zaitouna Alhamany

**Affiliations:** 1 Department of Pathology, Gyneco-Obstetric and Pediatric Hospital, Ibn Sina University Hospital, Rabat, Morocco; 2 Faculty of Medicine and Pharmacy, Mohammed V University of Rabat, Rabat, Morocco

**Keywords:** Angiosarcoma, Breast, Pathology, Case report

## Abstract

**Background:**

Angiosarcoma of the breast is a rare tumor, which may be primary or secondary to breast surgery or irradiation. It is characterized by polymorphic and nonspecific clinical and radiological features. A pathologist plays a key role in positive and differential diagnosis and in establishing the prognosis: only a histological examination can confirm the diagnosis, and the histologic grade is the most important prognostic factor. In fact, angiosarcomas of the breast constitute a very heterogeneous group and they are classified into three grades based on the degree of differentiation. We will illustrate diagnostic challenges through this new case of primary angiosarcoma of the breast. Microscopic findings were initially interpreted as a benign vascular tumor. We will also discuss the relevant medical literature.

**Case presentation:**

A 56-year-old Arabian woman presented with a palpable right breast mass that had been enlarging for 2 months, measuring 5 cm, without axillary lymphadenopathy. She had no personal or family history of breast surgery or breast irradiation. A mammography showed no evidence of spiculation. No suspicious calcifications were seen. A needle core biopsy was performed. Microscopic findings were initially interpreted as a benign vascular tumor. However, as the mass measured 5 cm, the diagnosis of angiosarcoma was more appropriate, and mastectomy without axillary dissection was performed. Microscopic examination found mild to moderately scattered pleomorphic cells, and scattered mitotic figures. It also showed papillary formations, solid foci of spindle cells, and hemorrhagic necrosis. The margins of the tumor were infiltrative. The diagnosis of primary intermediately differentiated angiosarcoma of the breast (grade II) was made. No distant metastases were found. Our patient was lost to follow-up and further treatment after mastectomy until she developed local tumor progression 4 months later.

**Conclusions:**

Through this case report, we emphasize the importance of clinicopathological confrontation in angiosarcoma of the breast.

## Background

Angiosarcoma of the breast is a rare tumor. It is defined by the World Health Organization as a malignant tumor showing endothelial differentiation [1]. It can be either primary or secondary to breast tissue irradiation or chronic lymphedema after mastectomy [1–4]. It is characterized by polymorphic and nonspecific clinical and radiological features causing diagnostic errors. Thus, a pathologist plays a key role in positive and differential diagnosis. A pathologist also plays a major role in establishing the prognosis by determining the grade. In fact, angiosarcomas of the breast are a heterogeneous group in which the grade is the most important prognostic factor. We will illustrate diagnostic challenges through this new case of primary angiosarcoma of the breast. Microscopic findings were initially interpreted as a benign vascular tumor. We will also discuss the relevant medical literature.

## Case presentation

**Fig. 1:**
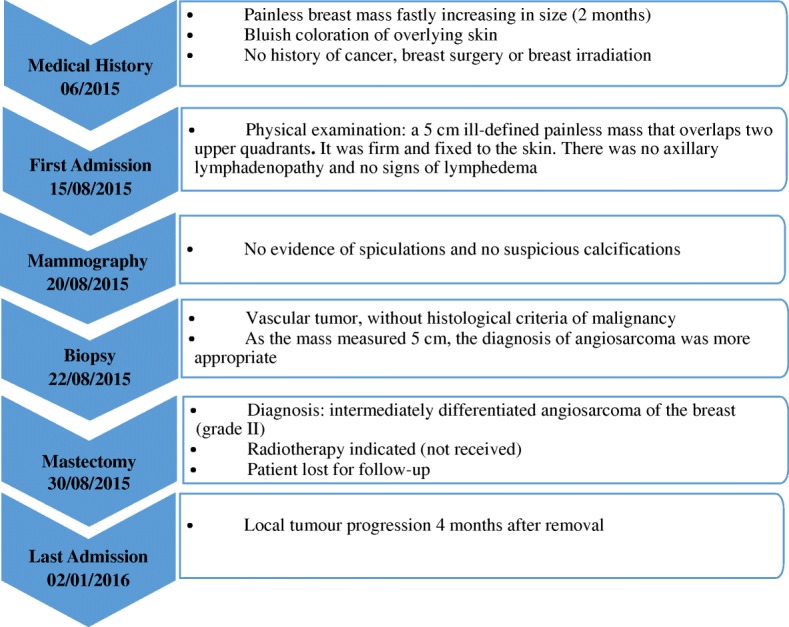
Case report timeline

**Fig. 2:**
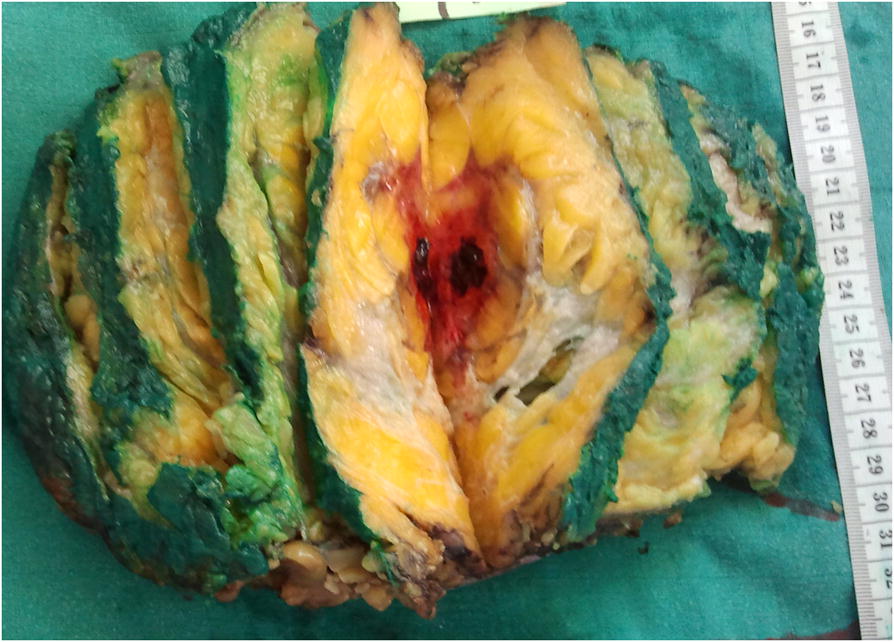
Macroscopic appearance of the breast tumor: It is an ill-defined mass with hemorrhagic appearance

**Fig. 3:**
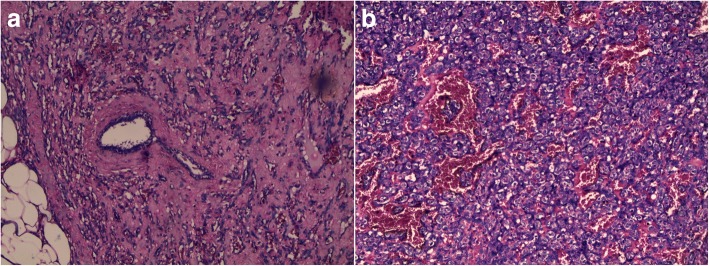
Representative micrograph of the breast tumor. Anastomosing vascular channels contain erythrocytes. Hematoxylin-eosin; **a** × 100, **b** × 200

**Fig. 4:**
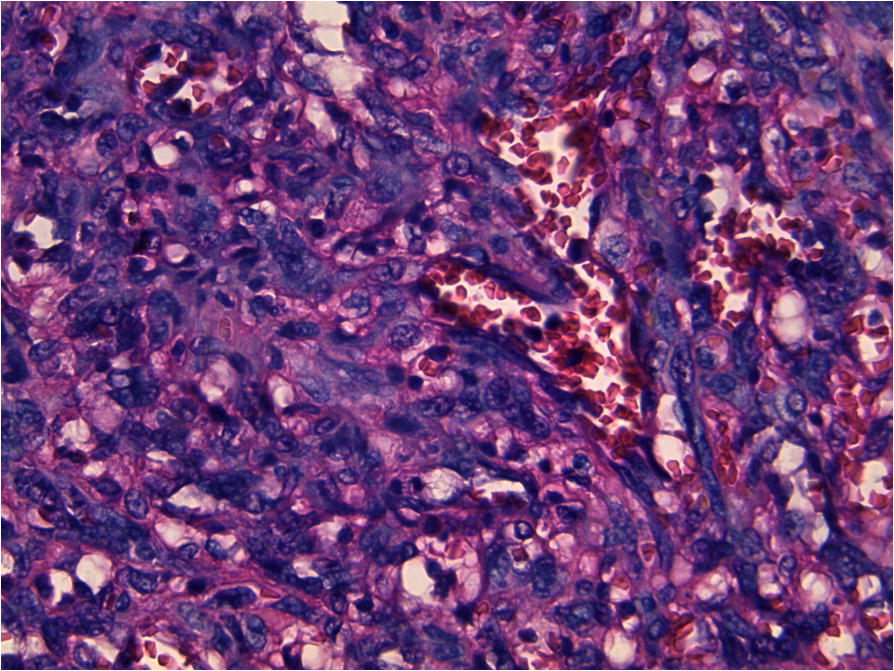
Representative micrograph of the breast tumor. Vascular channels are lined by atypical endothelial cells with hyperchromatic, spindle-shaped or round nuclei (hematoxylin-eosin × 400)

**Fig. 5:**
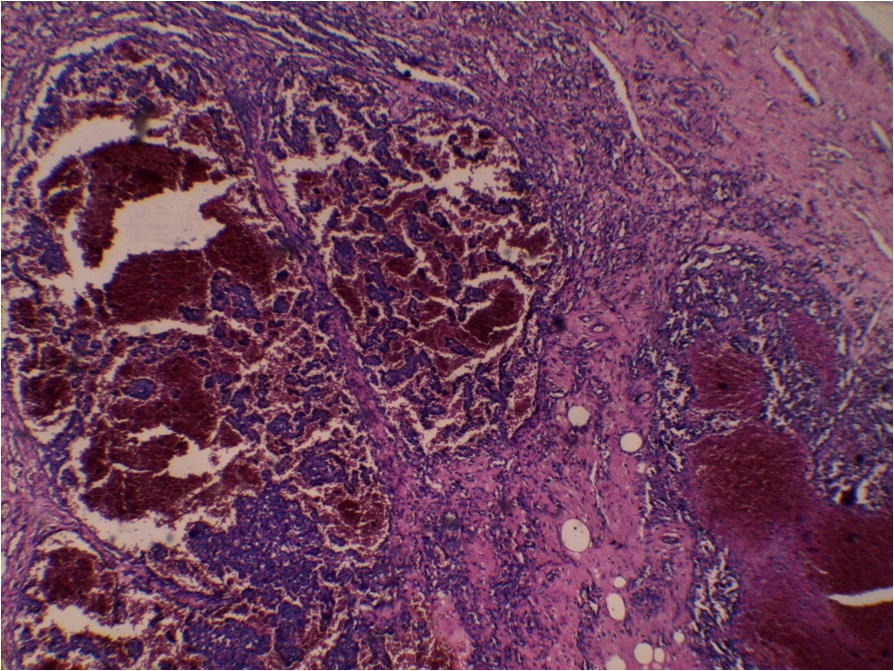
Representative micrograph of the breast tumor. There are papillary formations (hematoxylin-eosin × 100)

**Fig. 6:**
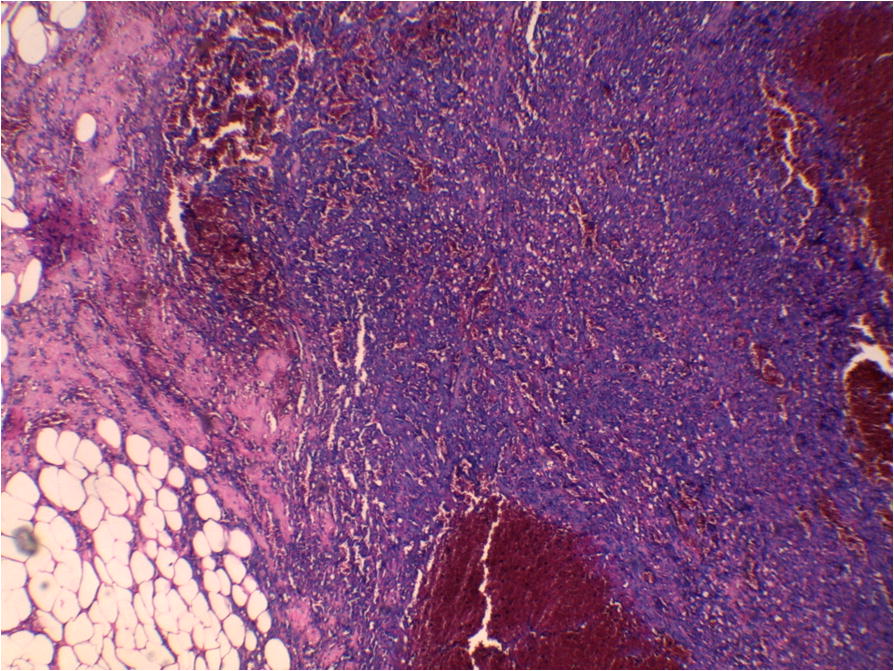
Representative micrograph of the breast tumor. There are also solid areas of spindle cells which invade adipose tissue (hematoxylin-eosin × 40)

**Fig. 7:**
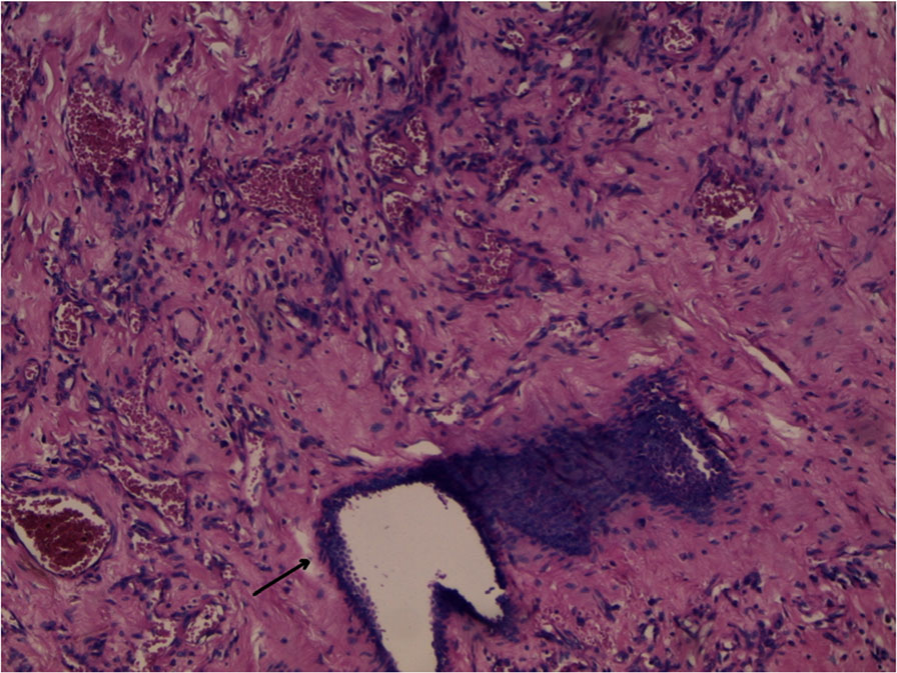
Anastomosing vascular channels in dense collagenous stroma engulfing breast elements (*arrow*). Hematoxylin-eosin × 100

A 56-year-old Arabian woman presented to our institution with a palpable right breast mass (Fig. 1). Two months prior to presentation she complained of a painless right breast lump that quickly increased in size with bluish coloration of overlying skin. She had no personal or family history of cancer, breast surgery, or breast irradiation. A physical examination revealed a 5 cm ill-defined painless mass that overlapped two upper quadrants. The mass was firm and fixed to the skin which was bluish without ulceration. There was no nipple retraction, no axillary lymphadenopathy, and no signs of lymphedema. A mammography showed no evidence of spiculation. No suspicious calcifications were seen. A needle core biopsy was performed and showed anastomosing round-to-oval spaces which contained erythrocytes. Lining cells had thin, elongated but hyperchromatic nuclei, which sometimes protruded into the luminal spaces. The neoplastic vascular channels invaded adipose tissue. Immunohistochemical stains performed on the core biopsy revealed membranous reactivity of the tumor cells for CD31-related antigen and CD34-related antigen. These findings were initially interpreted as a benign vascular tumor. However, as the mass measured 5 cm, the diagnosis of angiosarcoma was more appropriate. A mastectomy without axillary dissection was performed since angiosarcoma was suspected. At gross examination, the tumor appeared ill-defined, spongy, and soft (Fig. 2). A microscopic examination revealed vascular channels lined by atypical endothelial cells with hyperchromatic, spindle-shaped or round nuclei (Fig. 3). There were mild to moderately scattered pleomorphic cells, and scattered mitotic figures (Fig. 4). Other sections showed papillary formations, solid foci of spindle cells, and hemorrhagic necrosis (Figs. 5 and 6). The margins of the tumor were infiltrative (Fig. 7). The diagnosis of primary intermediately differentiated angiosarcoma of the breast (grade II) was made. No distant metastases were found. She was lost to follow-up and further treatment after mastectomy until she developed local tumor progression 4 months later.

## Discussion and conclusions

In terms of breast cancer, invasive carcinoma is by far the most common type. Angiosarcoma of the breast is a rare tumor; it accounts for 0.04% of all primary malignancies of the breast [1, 2, 5–7]. In our case, there was no axillary lymphadenopathy. The mammography showed no evidence of spiculation or suspicious calcifications. A biopsy showed anastomosing vascular spaces. Lining cells were almost bland. These results can be interpreted as a benign lesion. However, this did not correlate with the radiologic findings: 5 cm mass with ill-defined margins.

It may be primary or secondary to breast surgery or irradiation. Primary (*de novo*) angiosarcoma usually occurs in younger women [2, 5, 8] with an average age of 40 years [1, 8] compared to 67.5 years in secondary angiosarcoma [8]. The factors involved in carcinogenesis of primary angiosarcoma are exposure to vinyl chloride, arsenic and Thorotrast (thorium dioxide), chronic irritation induced by a foreign body, and local trauma [5]. Secondary angiosarcoma may occur in the skin and chest wall following radical mastectomy and local radiotherapy, and in the skin and breast parenchyma following breast conserving surgery and radiotherapy [1]. Furthermore, secondary angiosarcoma may occur in the skin and soft tissues of the arm following radical mastectomy and subsequent lymphedema (axillary dissection) [1].

It is manifested by a painless mass [2, 5, 6], sometimes with a pulsating character [5]. The overlying skin may have a bluish red discoloration, and there is no nipple retraction [2]. Axillary lymph node involvement is uncommon (0 to 5% of cases) [5] or even absent [2]. Mammography shows large, dense, and homogeneous mass, with sharp and sometimes polylobated contours [7]. There are no calcifications [5, 7] or spiculation which are often seen in breast carcinomas [2, 5]. On sonography, angiosarcoma appears as a heterogeneous lesion [5, 7] with both hyperechoic and hypoechoic appearance [2, 7]. Hypoechoic areas represent hemorrhagic or necrotic changes [7]. Color Doppler sonography reveals marked hypervascularity of the lesion [5, 7]. This hypervascular character is confirmed by computed tomography and magnetic resonance imaging with intravenously administered contrast injection [7]. The signal on T2-weighted image is hyperintense [2, 5, 7], suggesting the presence of vascular channels containing slow flowing blood [2].

The tumors are deeply located in the breast parenchyma [1]. Size is always > 2 cm [5] and usually > 4 cm [2]. It is reported that 12% of patients present with diffuse breast enlargement [1].

In some cases, diagnosis by fine-needle aspiration cytology and needle core biopsy may be difficult. Chen *et al.* reported a percutaneous biopsy false-negative rate of 37% [9]. Given the vascular nature of these tumors, macrobiopsy is often difficult to perform, hence the need for surgical resection [2].

On macroscopic examination, angiosarcomas have a spongy hemorrhagic appearance with ill-defined borders [1]. Poorly differentiated tumors appear as a solid fibrous lesion [1].

Only a histological examination can confirm the diagnosis. Three groups are defined according to the classification proposed by Donnell *et al*. [10]. Grade I (well differentiated) contains open anastomosing vascular channels invading the breast fat and parenchyma. A single layer of endothelial cells lines these channels. The nuclei of the endothelial cells may be hyperchromatic. Solid areas of spindle cells, hemorrhage (known as “blood lakes”), and necrosis are not present. In grade II (intermediately differentiated) 75% of the tumor is composed of the well-differentiated pattern seen in grade I, but there are additional solid cellular foci or papillary formations scattered throughout the tumor. Slightly increased mitotic activity is present. In grade III, solid areas of spindle cells and papillary formations are prominent. Mitoses are common. Areas of hemorrhage and necrosis are also seen.

The constituent cells show immunoreactivity for endothelial markers: CD31, CD34, and factor VIII [1, 6]. CD31 remains the most sensitive and the most specific endothelial cell marker. The role of immunohistochemical study is to confirm the vascular nature of tumor proliferation.

For grade I and II breast angiosarcoma, the differential diagnosis includes intramammary hemangioma, angiomatosis, and pseudoangiomatous stromal hyperplasia (PASH) [4], especially in a core biopsy. Hemangioma is usually 2 cm or less and is sharply defined [4]. Angiomatosis is a diffuse angioma with hemangioma and lymphangioma-like channels growing diffusely in breast tissue but sparing lobules and without nuclear atypia. Unlike angiomatosis, angiosarcoma infiltrates and dissociates breast lobules [4]. PASH is a benign lesion comprising stromal myofibroblastic proliferation and having the appearance of anastomosing slit-like pseudovascular spaces lined by spindle-shaped cells [11]. These spaces do not contain red cells, and have a perilobular concentric arrangement with a densely collagenous stroma [4]. There is no destruction of the normal breast tissue, no necrosis, and no fat invasion [11]. The myofibroblasts in PASH are positive for CD34 but are negative for factor VIII and CD31 [4, 11]. The differential diagnosis of grade I and II breast angiosarcoma is summarized in Table 1.

**Table 1:** Differential diagnosis of grade I (well-differentiated) and grade II (intermediately differentiated) angiosarcomas of the breast

Lesion	Grade I angiosarcoma	PASH	Hemangioma	Angiomatosis
Size	**Always > 2 cm**	Variable	**< 2 cm**	Diffuse
Anastomosing spaces	True vascular channels contain erythrocytes	**Pseudovascular spaces, without erythrocytes**	True vascular channels contain erythrocytes	True vascular channels contain erythrocytes
Destruction of adjacent breast tissue, invasion of fat	Yes	No	No	No
Dense hyaline stroma	Absent	**Present**	Absent	Absent
Lining cells	**Atypical with prominent and hyperchromatic nuclei**	Without atypia (very rarely atypia and hyperchromasia)	Without atypia	Without atypia
Factor VIII and CD31	Positive	**Negative**	Positive	Positive

*PASH* pseudoangiomatous stromal hyperplasia

Data in boldface: characteristic criteria for the differential diagnosis

For grade III, sarcomatoid carcinoma and other types of high-grade sarcomas should always be considered in the differential diagnosis, hence the necessity of cytokeratin antibodies and endothelial markers [4]. The differential diagnosis of poorly differentiated angiosarcoma is summarized in Table 2.

**Table 2:** Differential diagnosis of grade III (poorly differentiated) angiosarcoma of breast

	Grade III angiosarcoma	Sarcomatoid carcinoma	Other type of high-grade sarcomas
Endothelial markers	**Positive**	Negative	**Negative**
Cytokeratin	Negative	**Positive**	**Negative**

Data in boldface: characteristic criteria for the differential diagnosis

Angiosarcoma is an aggressive malignancy with high recurrence rates and poor overall survival [12]. According to Rosen and colleagues’ study [13], grade is an important prognostic factor. In fact, the 5 years disease-free survival rate was 76%, 70%, and 15% for grade I, grade II, and grade III tumors, respectively. Primary tumor size was not significantly related to the risk of recurrence or to survival. In contrast, Zelek *et al.* [14], by reviewing eight cases of breast angiosarcoma, found that the tumor size was correlated with the rate of disease-free survival at 10 years. The prognosis was even worse when the tumor size was greater than 10 cm. Primary angiosarcoma of the breast gives metastasis mainly to lungs, liver, bones, skin, and the contralateral breast [1, 2].

There are no treatment standards for breast angiosarcomas because of their rarity [2]. However, treatment is primarily surgical. Mastectomy with negative margins is the recommended treatment [2, 5], without axillary dissection [5, 6]. Complementary radiation therapy is necessary in case of tumorectomy [5]. Adjuvant radiation and chemotherapy improve survival outcomes in patients with tumor size > 5 cm [14, 15]. Chemotherapy is beneficial in high-grade lesions [3, 6] and in the metastatic setting [3]. Recently, with the exploitation of vascular endothelial growth factor (VEGF)-A and VEGF-C and the receptor VEGF-R1, anti-angiogenic treatment is certainly a highly promising therapeutic approach [2, 5].

Through this case report, we emphasize the importance of clinicopathological confrontation in angiosarcoma of the breast. This is especially true in cases of biopsy showing a well-differentiated angiosarcoma. It poses a problem of differential diagnosis with benign vascular pathology. It has a poor prognosis and presents therapeutic management difficulties.

## Abbreviations

PASH: Pseudoangiomatous stromal hyperplasia
VEGF: Vascular endothelial growth factor
